# Structural Magnetic Resonance Imaging (MRI) in Differentiating Radionecrosis from Metastatic Recurrence in Post-Surgical Patients: A Tertiary Center Experience

**DOI:** 10.3390/cancers18213439

**Published:** 2026-10-24

**Authors:** Flavia Bino, Camilo Pineda, José Pariente, Solanche Jasmin Santillan-Zorrilla, Juan Andrés Mora, Izaskun Valduvieco Ruiz, Yensa Rodríguez, Iban Aldecoa, Guillem Martín Vidal, Albert Calvo Porcel, Núria Bargalló, Sofía González-Ortiz

**Affiliations:** 1 Department of Neuroradiology, Fondazione IRCCS San Gerardo dei Tintori, Via Pergolesi 33, 20900 Monza, Italy; 2 School of Medicine, University of Milano-Bicocca, Piazza dell’Ateneo Nuovo 1, 20126 Milano, Italy; 3 Neuroradiology Section, Department of Radiology, Diagnostic Image Center, Hospital Clinic of Barcelona, University of Barcelona, 08036 Barcelona, Spain; cpineda@clinic.cat (C.P.); jamoras@clinic.cat (J.A.M.); yerodri@clinic.cat (Y.R.); gumartin@clinic.cat (G.M.V.); calvoa@clinic.cat (A.C.P.); bargallo@clinic.cat (N.B.); sgonzal@clinic.cat (S.G.-O.); 4 Fundació de Recerca Clínic Barcelona, Institut d’Investigacions Biomèdiques August Pi I Sunyer, 08036 Barcelona, Spain; pariente@recerca.clinic.cat; 5 Department of Radiation Oncology, Hospital Clínic de Barcelona, University of Barcelona, 08036 Barcelona, Spain; santillan@clinic.cat (S.J.S.-Z.); ivalduvi@clinic.cat (I.V.R.); 6 Department of Pathology, Biomedical Diagnostic Center (CDB), Hospital Clínic de Barcelona, University of Barcelona, 08036 Barcelona, Spain; ialdecoa@clinic.cat

**Keywords:** brain metastasis, magnetic resonance imaging, radionecrosis, radiotherapy

## Abstract

**Purpose**: Distinguishing radionecrosis (RN) from recurrent brain metastasis (rM1) remains a major challenge in the follow-up of patients treated with surgery and radiotherapy. Although advanced imaging techniques have been proposed to improve diagnostic accuracy, they are not always available in routine clinical practice and may be limited by postoperative changes and artifacts. Histopathologically validated studies in this setting remain scarce. **Methods**: We retrospectively evaluated 16 patients with 17 histologically confirmed lesions who underwent repeat surgery for suspected progression after the resection of brain metastases and adjuvant radiotherapy. Preoperative MRI examinations were reviewed by two neuroradiologists using a standard MRI protocol. Qualitative features included signal characteristics, T1/T2 mismatch, enhancement patterns, and the presence of the “inflammatory cloud” and “incomplete ring enhancement” signs. Quantitative analysis included lesion, edema and necrosis volumes, apparent diffusion coefficient (ADC), intratumoral susceptibility signal (ITSS) grading, and volumetric lesion quotient (vLQ). A LASSO logistic regression model with leave-one-out cross-validation and bootstrap stability analysis was used to identify the most relevant predictors. **Results**: Histopathology demonstrated 11 RN lesions and six recurrent metastases. The LASSO model achieved an area under the curve of 0.71 and an overall accuracy of 76% (13/17 lesions) with a sensitivity of 82% for RN and a specificity of 67% for rM1. RN lesions were characterized by significantly higher ADC values and ITSS grades and by the exclusive presence of the inflammatory cloud and incomplete ring enhancement signs. Stability analysis identified incomplete ring enhancement (49%), ITSS grade (45%), ADC values (45%), and inflammatory cloud (44%) as the most robust predictors of RN, whereas volumetric measurements showed limited independent predictive value. **Conclusions**: In patients with histologically confirmed post-treatment lesions, conventional MRI provided several reproducible features that aided differentiation between radionecrosis and recurrent brain metastasis. The inflammatory cloud, incomplete ring enhancement, elevated ADC values, and higher ITSS grades emerged as the most robust imaging markers of RN. While predictive multivariable models remain exploratory, these readily available MRI features may improve diagnostic confidence in routine clinical practice and warrant validation in larger multicenter cohorts.

## Simple Summary

After the treatment of brain metastases, new or enlarging abnormalities on magnetic resonance imaging may represent either radiotherapy-related tissue damage, especially radionecrosis, or tumor recurrence. Distinguishing these two conditions is essential as they require different management strategies; however, this remains challenging in clinical practice. In this study, we investigated whether routinely available magnetic resonance imaging features could help address this diagnostic problem. Our findings identified a combination of imaging characteristics associated with radionecrosis, suggesting that a careful evaluation of conventional magnetic resonance images may provide useful information without requiring advanced imaging techniques. These findings may help improve diagnostic confidence when assessing suspicious brain lesions after treatment. Further studies involving larger groups of patients are needed to confirm the usefulness of these imaging features in clinical practice.

## 1. Introduction

Brain metastases are the most common intracranial tumors in adults and a frequent complication in patients with locally advanced cancer, occurring in up to 30–40% during the course of their disease [1]. Contrast-enhanced magnetic resonance imaging (MRI) is the gold standard for diagnosis and managing brain metastases due to its higher spatial resolution compared with contrast-enhanced computed tomography (CT), which is primarily used in emergency settings [2].

The management of brain metastases has evolved toward multimodal approaches including surgery, radiotherapy, chemotherapy, and immunotherapy. Surgical resection is indicated for patients with a single accessible lesion and good performance status, which is often followed by adjuvant radiotherapy. In large lesions causing mass effect, radiotherapy options include whole-brain radiation therapy (WBRT) and stereotactic radiation therapy (SRT) [3]. WBRT is currently reserved for patients with multiple large unresectable metastases and in a supportive care scenario [4], but it carries risks of cognitive dysfunction and other long-term toxicities [3]. SRTs, including stereotactic radiosurgery (SRS) and fractionated SRS, have increasingly become the preferred approach for patients with a limited number of brain metastases because of its ability to deliver a highly focused radiation to the target while minimizing exposure of the surrounding healthy brain tissues. Fractionated SRS is increasingly used for larger brain metastases (>3 cm) or those located near critical structures, as allows normal tissue recovery [4].

SRTs are associated with acute (e.g., dermatitis, fatigue, headache, nausea and vomiting) and delayed reactions including pseudoprogression (PsP) and radionecrosis (RN), which represent opposite extremes along the spectrum of radiation-induced injury. Although sometimes used interchangeably, PsP and RN differ in timing, pathophysiology, histopathology, and prognosis, both resolving without treatment modification [5,6]. PsP usually occurs within weeks to months after treatment, particularly in patients receiving concomitant chemotherapy (≈20% incidence) and follows a self-limiting course [7], while RN is a delayed complication, arising 9 to 12 months post-treatment or even years later [5]. Its incidence varies widely, ranging from 0% to 30%, due to multiple factors, including radiation dose, re-irradiation, tumor size, prior WBRT, concomitant chemotherapy and specific histology subtypes.

The management and prognosis of patients with post-treatment changes (PsP or RN) differ significantly from M1 recurrence. RN can be treated with different strategies from a close monitoring to surgical debulking, which is reserved for symptomatic or medically refractory lesions or in an urgent setting [1,7]. In contrast, M1 recurrence implies disease progression and requires reassessment for further local or systemic treatment strategies.

MRI plays an essential role in the differentiation between recurrence and post-treatment changes. Radiologically, both entities can appear with the presence of new or enlarging enhancing areas with vasogenic edema within the radiation field, unfortunately, often mimicking each other and leading to potential misinterpretation and inappropriate management [7].

While advanced MRI techniques, such as perfusion imaging and spectroscopy, have been proposed to improve diagnostic accuracy [1,7,8], they are not always available in routine clinical practice, and they require longer acquisition time. Moreover, advanced sequences often yield to inconclusive results, particularly in the presence of calcification and hemorrhage, which are features that are commonly seen in post-surgical patients and RN that can complicate the interpretation of imaging findings and blur the distinction between RN and tumor recurrence.

Due to the increasing number of patients who undergo surgery followed by SRTs and novel chemotherapies, there is a critical need to identify reliable MRI features on standard sequences to guide radiologists in differentiating RN from tumor recurrence.

This study aimed to identify MRI features from standard imaging protocols that may help differentiate RN from metastatic recurrence in post-surgical patients, using histopathology as the reference standard.

## 2. Materials and Methods

### 2.1. Study Design

This retrospective study included patients with a diagnosis of brain metastases who underwent initial surgical resection followed by adjuvant radiotherapy. During follow-up, patients were referred for a second surgery due to suspected disease progression on MRI. Pre-second-surgery MRI scans performed between July 2019 and January 2024 were retrieved from our Picture Archiving and Communication System (PACS) for evaluation. Inclusion criteria were as follows: (i) availability of pre-surgical contrast-enhanced brain MRI; (ii) intra-axial lesions; and (iii) clear histological diagnosis. Histopathological diagnoses were established by the pathologists according to the European Association for Neuro-Oncology (EANO) consensus recommendations. Lesions showing treatment-related necrotic changes without viable tumor cells were classified as radionecrosis, whereas the presence of viable metastatic tumor cells defined recurrent metastasis. Cases with extensive mixed or equivocal histopathological findings were excluded [9]. Adjuvant radiotherapy included fractionated stereotactic radiotherapy (75%), stereotactic radiosurgery (19%), and whole-brain radiotherapy (6%). Detailed dosimetric parameters were not systematically captured in this retrospective imaging repository.

**Figure 1:**
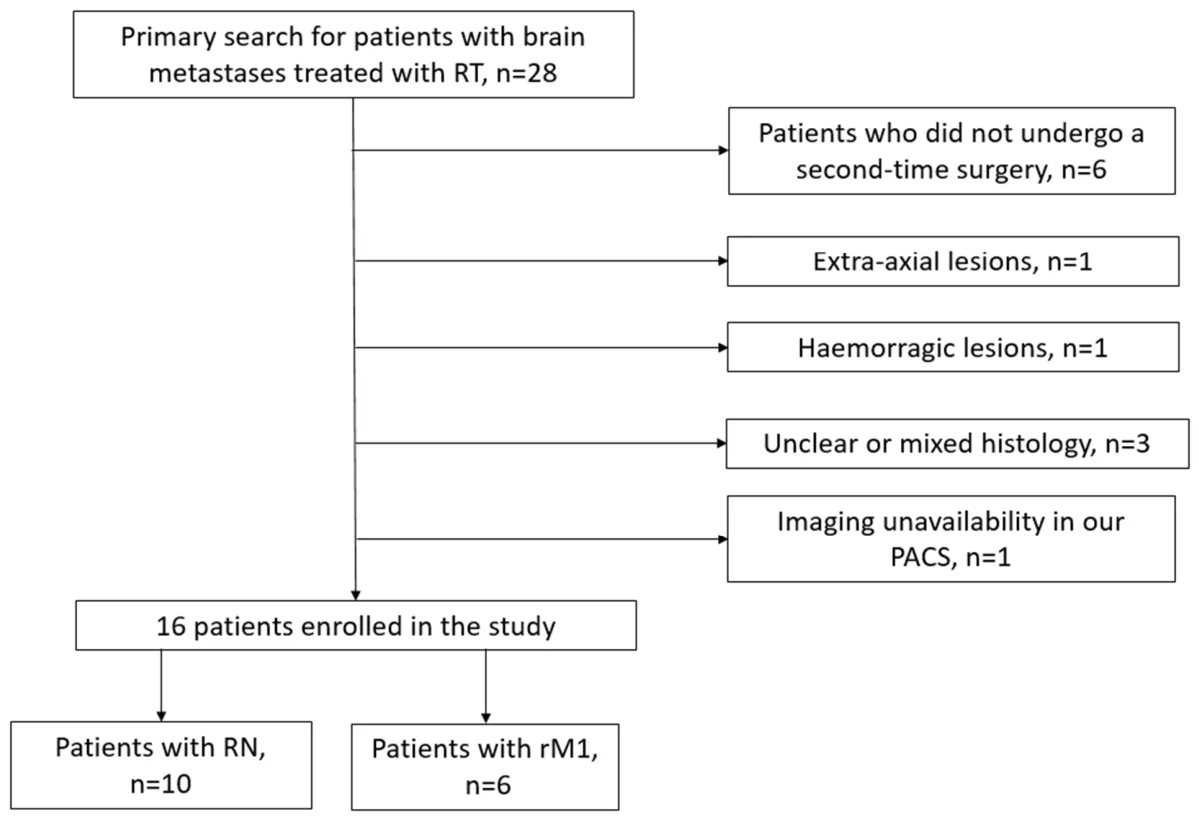
Flow chart of study population.

Exclusion criteria included (i) patients who did not undergo a second-time surgery; (ii) extra-axial lesions; (iii) completely hemorrhagic lesions; (iv) not definite histological diagnosis; and the (v) unavailability of MRI images in our PACS (Figure 1).

### 2.2. MRI Technique

MRI scans were performed using 1.5 T or 3 T scanners (Signa™, General Electrics (GE) Heathcare, Waukesha, WI, USA; MAGNETOM, Siemens Healthineers AG, Erlangen, Germany). 

The imaging protocol included the following sequences: 3D-T1-weighted (T1-w) Gradient Echo (GRE) (MPRAGE—Magnetization Prepared Rapid Gradient Echo Imaging), axial T2-weighted (T2-w) Fast Spin Echo (FSE), axial and coronal Fluid-Attenuated Inversion Recovery (FLAIR), axial Susceptibility Weighted Imaging (SWI) and axial Diffusion Weighted Imaging (DWI).

After contrast agent administration (0.2 mL/kg body weight, Clariscan™, Gadoteric acid, GE Healthcare), volumetric T1-weighted GRE (MPRAGE) and axial T1-weighted Spin Echo (SE) sequences were acquired.

#### 2.2.1. Imaging Analysis

All the images, available in PACS, were reviewed by two radiologists: a senior neuro-radiologist and a junior radiologist with one year of experience in neuroradiology. Both readers were blinded to the histopathological diagnosis, and discrepancies were resolved by consensus. The following qualitative characteristics were analyzed:

- Lesions’ signal intensity (SI) (rated as hyperintense, hypointense, or isointense) in different sequences (T1-w, T2-w, and FLAIR);
- T1/T2 mismatch, defined as the spatial discordance between the boundaries of the lesions on T2-w and post-contrast T1-w [7];
- Enhancement patterns (categorized as peripheral non-linear, peripheral/marginal, solid, heterogeneous);
- The “inflammatory cloud”, defined as a focal enhancement around the lesion with indistinct, cloudy-like borders [10];
- The “incomplete ring enhancement”, defined as a discontinuous enhancement around the lesion [10].

The following quantitative characteristics were evaluated:

- Volume of enhancing components;
- Volume of necrosis and extent of edema;
- Volumetric ADC values;
- Intratumoral susceptibility signal intensity (ITSS) grading;
- Volumetric lesion quotient (vLQ).

Enhancing components on post-contrast T1-w images, necrotic changes on T2-w images, and edema extension on FLAIR images were manually segmented and reported as volumes using a dedicated tool of the advanced radiology software Mint Lesion™ Research (version 3.10; mint Medical GmbH, Heidelberg, Germany).

ADC values were evaluated by co-registering the ADC maps with the post-contrast T1-w images using rigid 3D registration with normalized mutual information and trilinear interpolation to achieve a volumetric and comprehensive assessment of the variable. The mean ADC value was reported. The grading of ITSS, defined as low signal intensity and a fine linear or dot-like structure, with or without conglomeration, seen on SWI, was performed according to the previous definition. Lesions were categorized as follows: Grade 0 (no ITSSs), Grade 1 (1–5 ITSSs), Grade 2 (6–10 ITSSs) and Grade 3 (> 11 ITSSs) [11].

A vLQ, defined as the ratio of the volume of a definable nodule on T2-w and the volume of enhancing components on post-contrast T1-w, was calculated by manually segmenting well-defined hypointense nodules and enhancing lesions [12].

#### 2.2.2. Statistical Analysis

Both qualitative and quantitative imaging characteristics were evaluated and compared between the RN and rM1 groups. Qualitative variables were compared using Fisher’s exact test and the Chi-Squared test, the latter specifically applied to compare lesions’ signal intensity. Enhancement patterns were reported as percentages. Quantitative variables were compared using the Mann–Whitney U test due to non-normal distribution and were reported as median and interquartile range (IQR). A two-tailed *p*-value ≤ 0.05 was considered statistically significant. False discovery rate (FDR) adjustments using the Benjamini–Hochberg procedure were applied across univariate tests, reporting FDR q-values with q < 0.10 indicating exploratory significance.

To address the limitations of small sample size and high dimensionality, a penalized regression approach was subsequently applied. A LASSO logistic regression model (alpha = 1) was used for variable selection and shrinkage, while model performance was evaluated using LOOCV.

In each of the 17 folds, the model was trained on n–1 observations, and the optimal regularization parameter (lambda) was selected via internal cross-validation (lambda.min criterion).

The overall discriminative ability of the model was assessed by the area under the ROC curve (AUC). Ninety-five percent confidence intervals (95% CI) were calculated for sensitivity, specificity, accuracy, and AUC. Leave-one-patient-out cross-validation (LOPO-CV) was conducted to assess potential clustered lesion effects. The optimal classification threshold was determined using the Youden Index, maximizing the sum of sensitivity and specificity for the detection of radionecrosis.

To assess the robustness of the selected predictors, a stability selection analysis was performed using a stratified bootstrap procedure (B = 1.000 iterations) with resampling conducted within each class. A LASSO model was fitted in each iteration and selection stability was defined as the percentage of iterations in which each variable retained a non-zero coefficient.

## 3. Results

### 3.1. Study Population

Sixteen patients (10 females and 6 males: mean age 57 years) with a total of 17 brain lesions were included (as one patient presented two lesions). Histopathology revealed 11 RN and 6 rM1. Twelve patients (75%) had metastases from lung cancer, 2 (13%) from breast cancer, 1 (6%) from melanoma and 1 (6%) from colorectal cancer. RN was observed in patients with previously operated metastases lung cancer (8 cases, 50%) and breast cancer (2 cases, 12%). Twelve patients (75%) received fractionated SRS, 3 patients (19%) were treated with SRS and 1 (6%) with WBRT.

### 3.2. Qualitative Imaging Features

Qualitative characteristics are summarized in Table 1. A significant difference in T2SI was observed (raw *p* = 0.007, Benjamini–Hochberg FDR q = 0.072) with RN lesions more frequently appearing hyperintense compared to rM1. No significant differences were found in T1 signal intensity, although RN lesions tended to be more hypointense.

T1/T2 mismatch did not reach statistical significance (raw *p* = 0.11, FDR q = 0.164) but rM1 demonstrated higher concordance in lesion boundaries on T2-w and postcontrast T1w.

**Figure 2:**
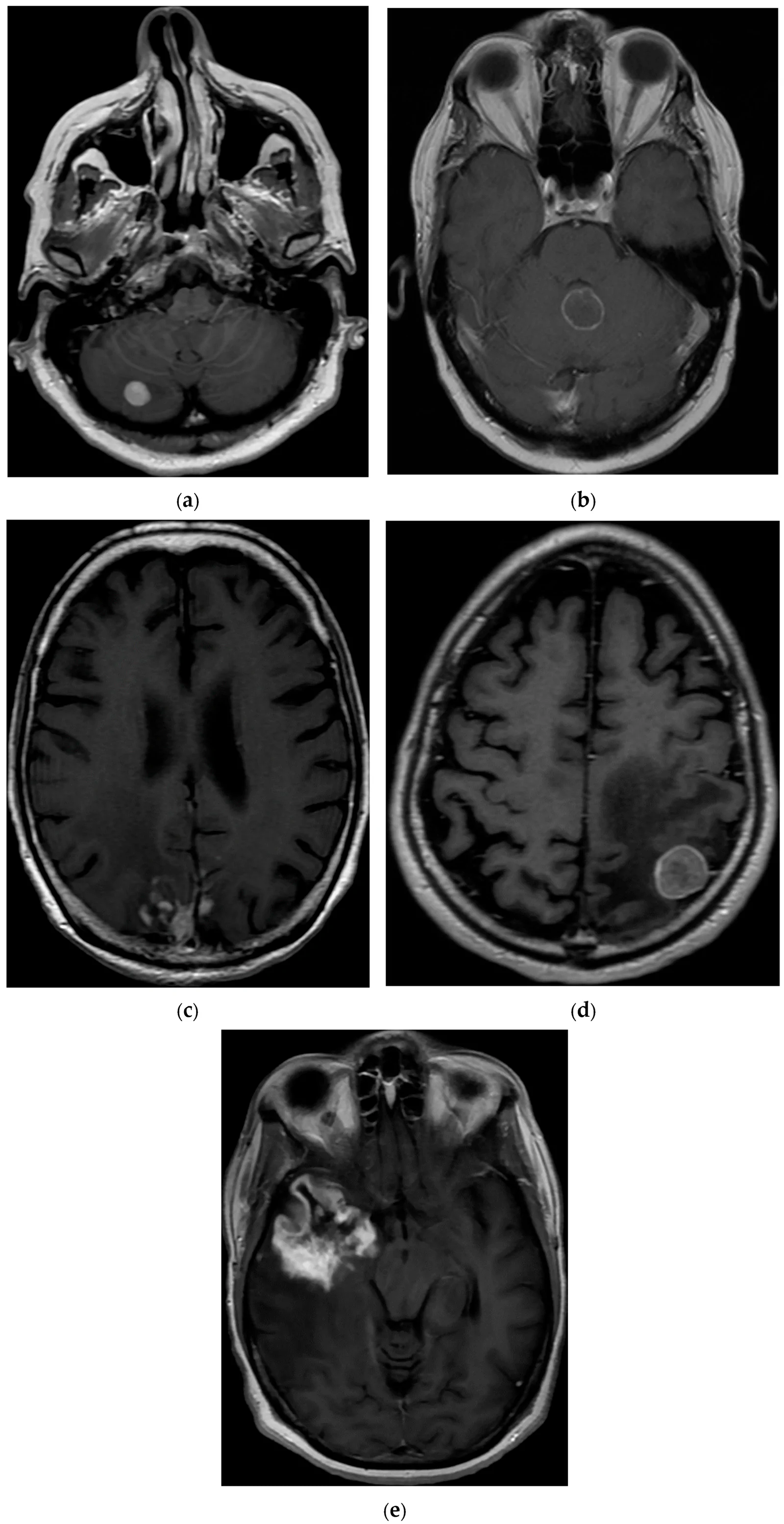
Examples of enhancement patterns. Homogeneous (**a**), peripheral (**b**), patchy (**c**), heterogeneous (**d**) and peripheral non-linear (**e**).

Significant differences (raw *p* = 0.034, FDR q = 0.072) were identified in the presence of the “inflammatory cloud” and the “incomplete ring enhancement”, both of which were exclusively identified in RN lesions. Peripheral non-linear enhancement was observed in RN, while rM1 displayed more variable enhancement patterns. Only one RN lesion shows a non-categorizable enhancement pattern, which was described as “patchy”. A detailed breakdown of enhancement patterns is provided in Table 2. Examples of enhancement patterns are shown in Figure 2.

### 3.3. Quantitative Imaging Features

Statistically significant differences were observed in ADC values (raw *p* = 0.036, FDR q = 0.072) and ITSS grading (raw *p* = 0.018, FDR q = 0.072). RN lesions exhibited higher values on ADC maps and a greater ITSS grading than rM1 ones. None of the RN lesions were classified as Grade 0 in ITSS evaluation. Volumetric LQ also differed significantly between the two groups with RN showing lower ratios compared to rM1 lesions. Although no statistically significant differences were found in the volumes of enhancing components, necrosis and edema, RN lesions generally tended to show larger volumes across these parameters. Further details of quantitative characteristics are provided in Table 3.

**Figure 3:**
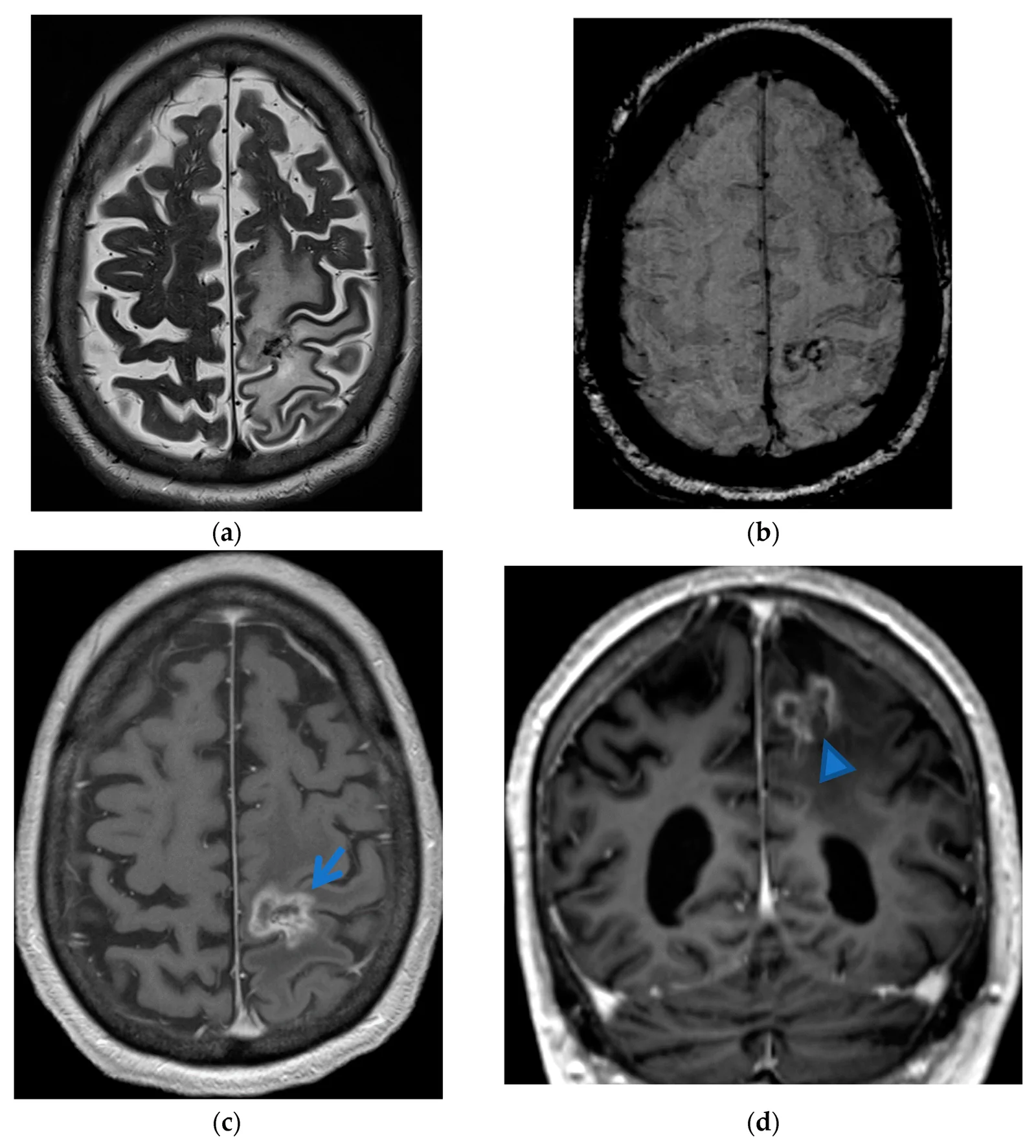

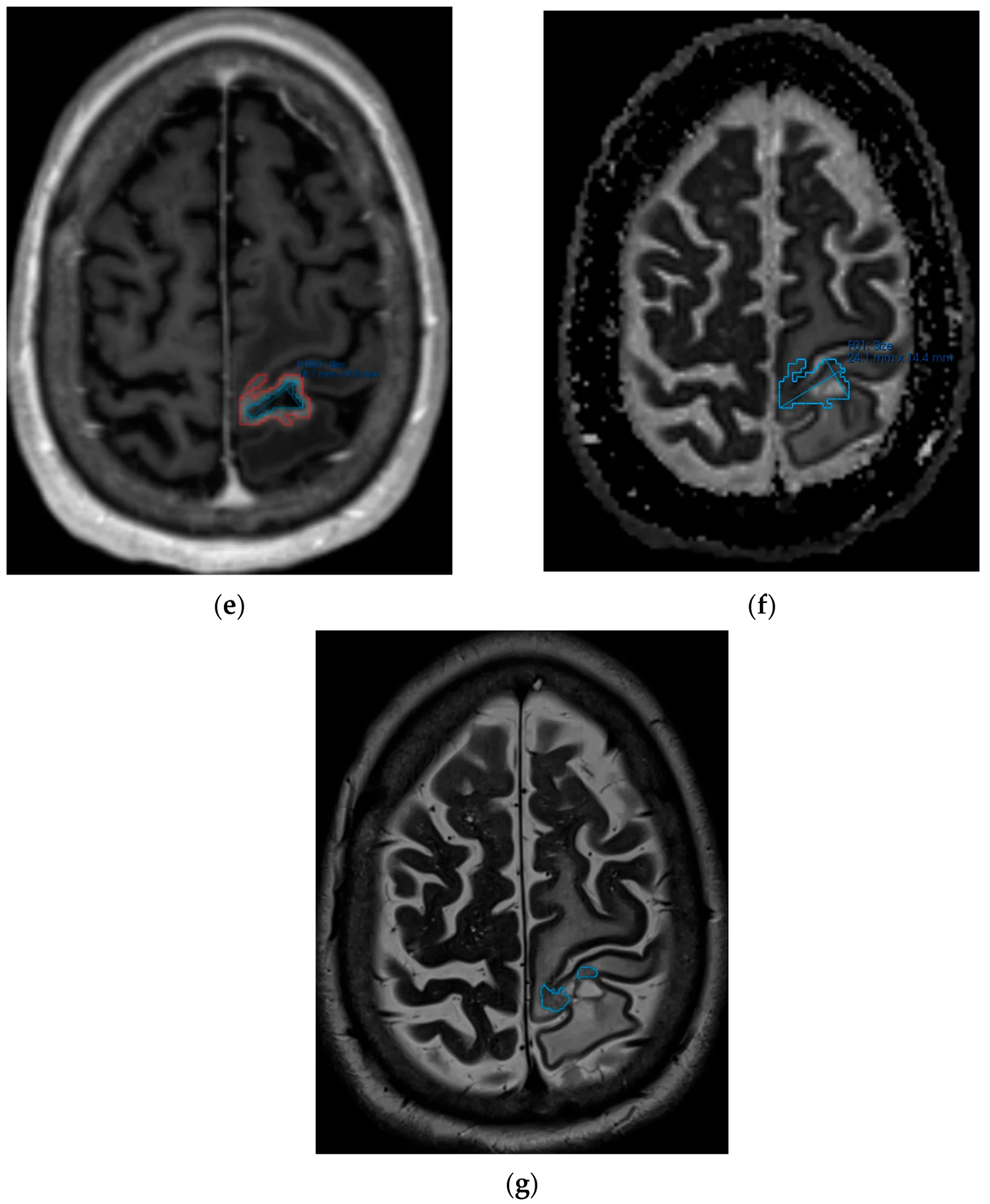
A poorly delineated mass in the left parietal lobe, which was confirmed as RN. Axial T2-weighted FSE (**a**) shows an ill-defined mass with perilesional edema and a Grade 2 ITSS on SWI (**b**) and inhomogeneous contrast enhancement (**c**,**d**). Axial and coronal post-contrast T1-weighted GRE images reveal a focal, poorly defined enhancement around the lesion, described as an “inflammatory cloud” (blue arrow in **c**), along with an incomplete ring enhancement on the inferior border of the lesion (blue arrow head in **d**). Manual segmentation of the lesion was performed on post-contrast T1-weighted (**e**), ADC map (**f**), and T2w (**g**) to evaluate ADC values and calculate the vLQ comprehensively. In this case, the vLQ was 0.3, indicating a lack of significant solid components within the lesion.

**Figure 4:**
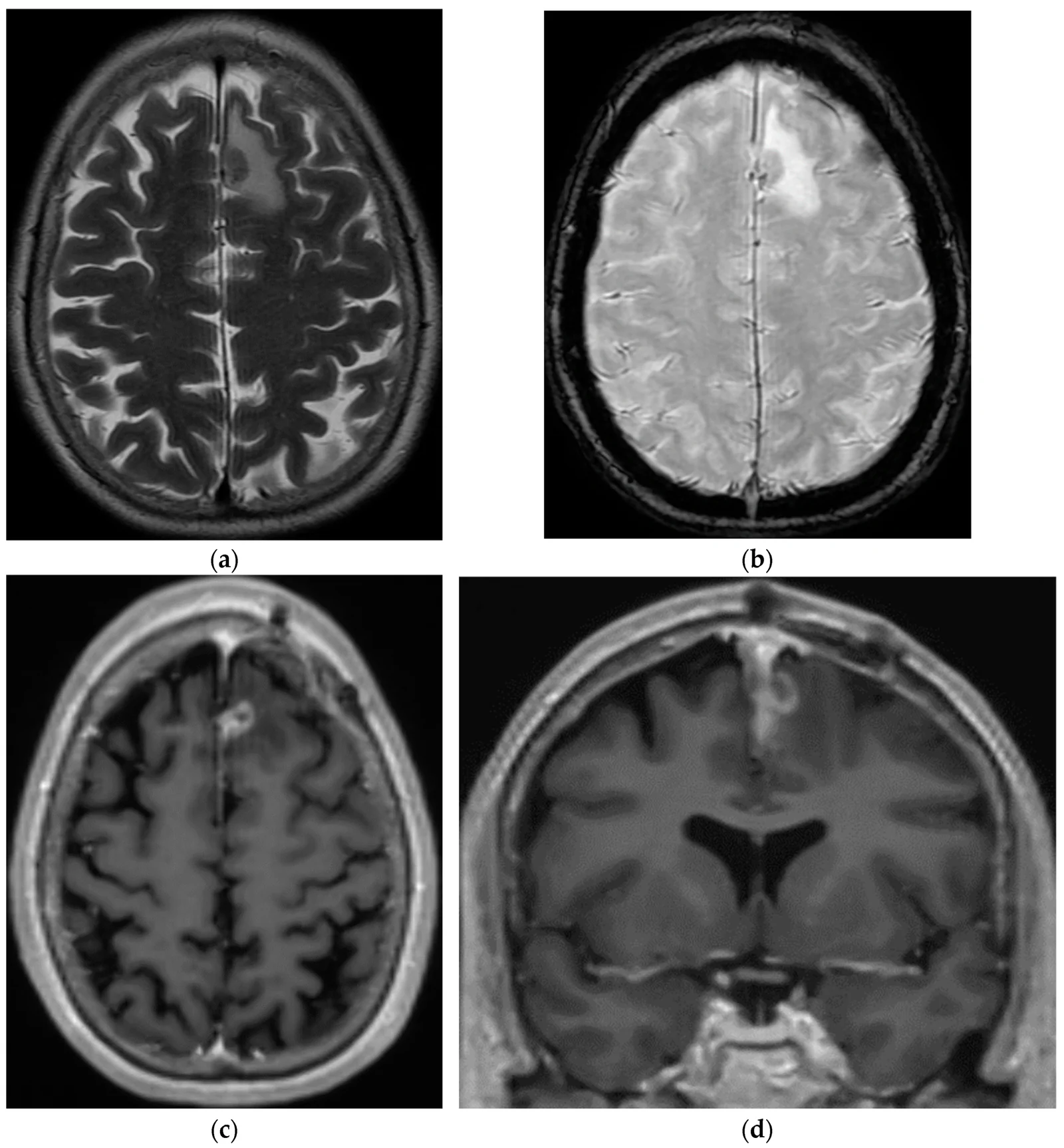

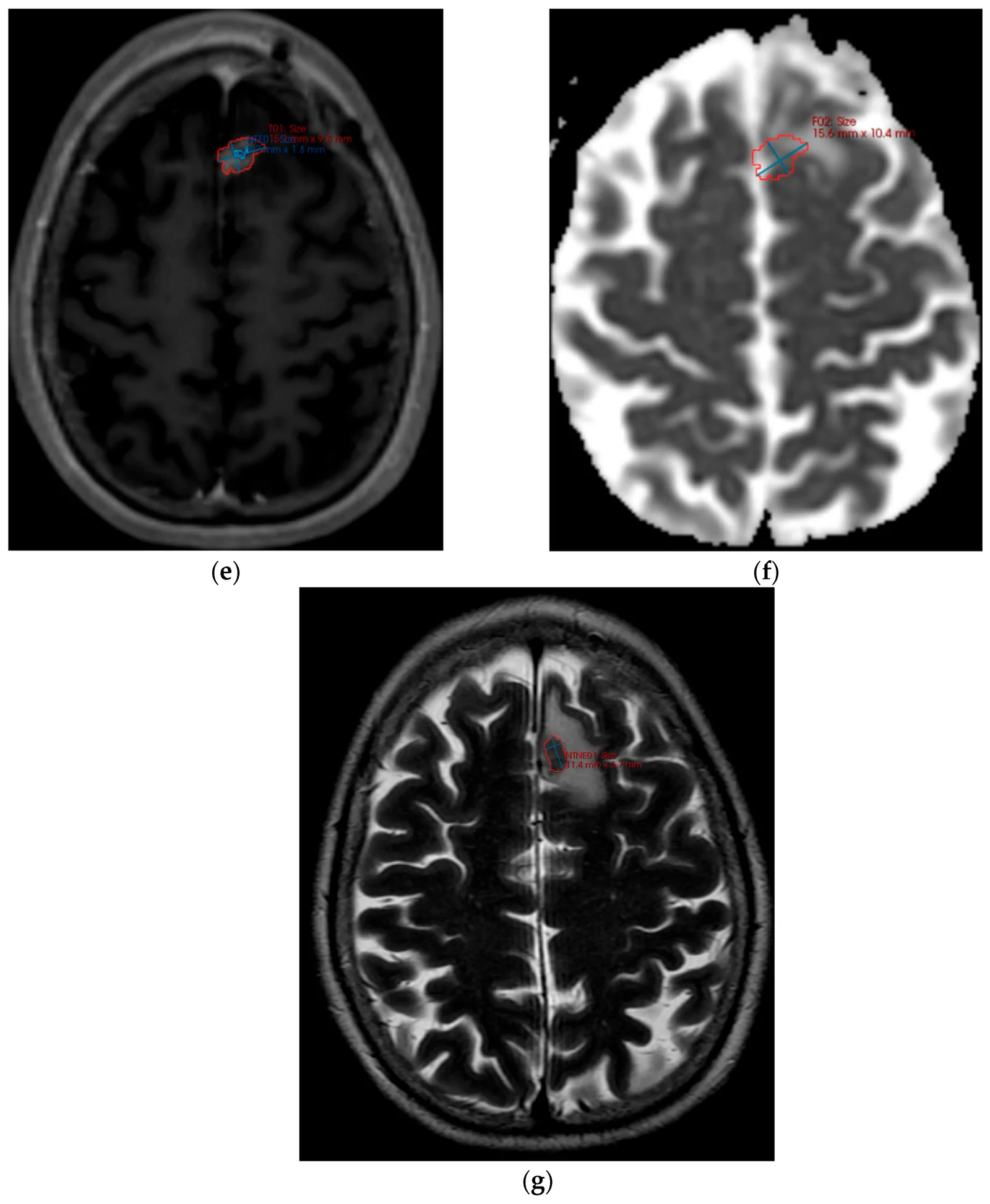
A well-defined lesion in the left frontal lobe, which was proven to be a rM1. Axial T2-weighted TSE (**a**) demonstrates a solid mass with clearly defined borders, mild perilesional edema, and no evidence of ITSS on SWI (**b**). Axial and coronal post-contrast T1w GRE (**c**,**d**) reveal a complete, linear peripheral enhancement. Manual segmentation was performed on post-contrast T1-weighted (**e**), ADC map (**f**), and T2w (**g**) to evaluate ADC values and calculate the vLQ. In this case, a vLQ of 0.5 was determined, reflecting a higher solid component compared to an RN lesion.

Figure 3 and Figure 4 show an example of significant qualitative and quantitative differences between RN and rM1, respectively.

### 3.4. Predictive Modeling and Feature Stability Analysis Quantitative Imaging Features

**Figure 5:**
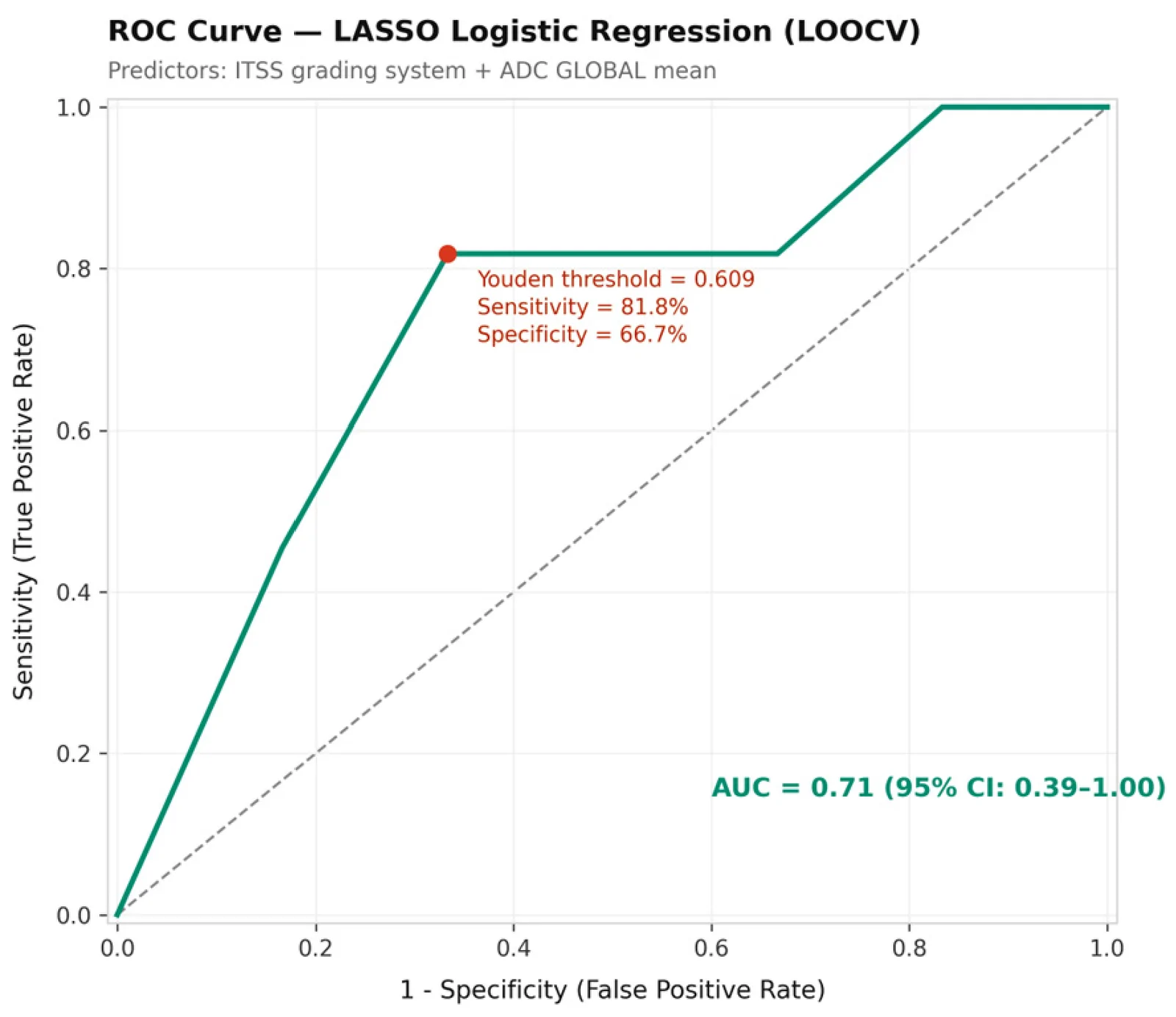
ROC curve of the LASSO model validated with LOOCV. It shows an exploratory discriminative performance (AUC-ROC = 0.71, 95% CI: 0.39–1.00; step-curve AUC = 0.73). The optimal Youden threshold (0.609) provides a sensitivity of 81.8% (95% CI: 52.3–94.9%) and a specificity of 66.7% (95% CI: 30.0–90.3%), indicating better performance in identifying radionecrosis than recurrent metastasis. The irregular shape of the curve likely reflects the small sample size and the variability introduced by LOOCV.

The LASSO model evaluated via LOOCV yielded an AUC-ROC of 0.71 (95% CI: 0.39–1.00), demonstrating a moderate discriminative performance. Using the optimal threshold defined by the Youden Index (0.61), an overall accuracy of 76.5% (95% CI: 52.7–90.4%) (13/17 cases correctly classified) was achieved (Supplementary Table S1). Sensitivity for radionecrosis was 81.8% (9/11) [95% CI: 52.3–94.9%], while specificity for recurrent metastasis was 66.7% (4/6) [95% CI: 30.0–90.3%]. The confusion matrix showed two false negatives and two false positives, indicating a balanced classification performance despite class imbalance. In the initial LOOCV folds, the ITSS grading system and ADC values were the most frequently selected predictors, both showing positive associations with RN (Figure 5).

The stratified bootstrap analysis further assessed the stability of these predictors.

**Figure 6:**
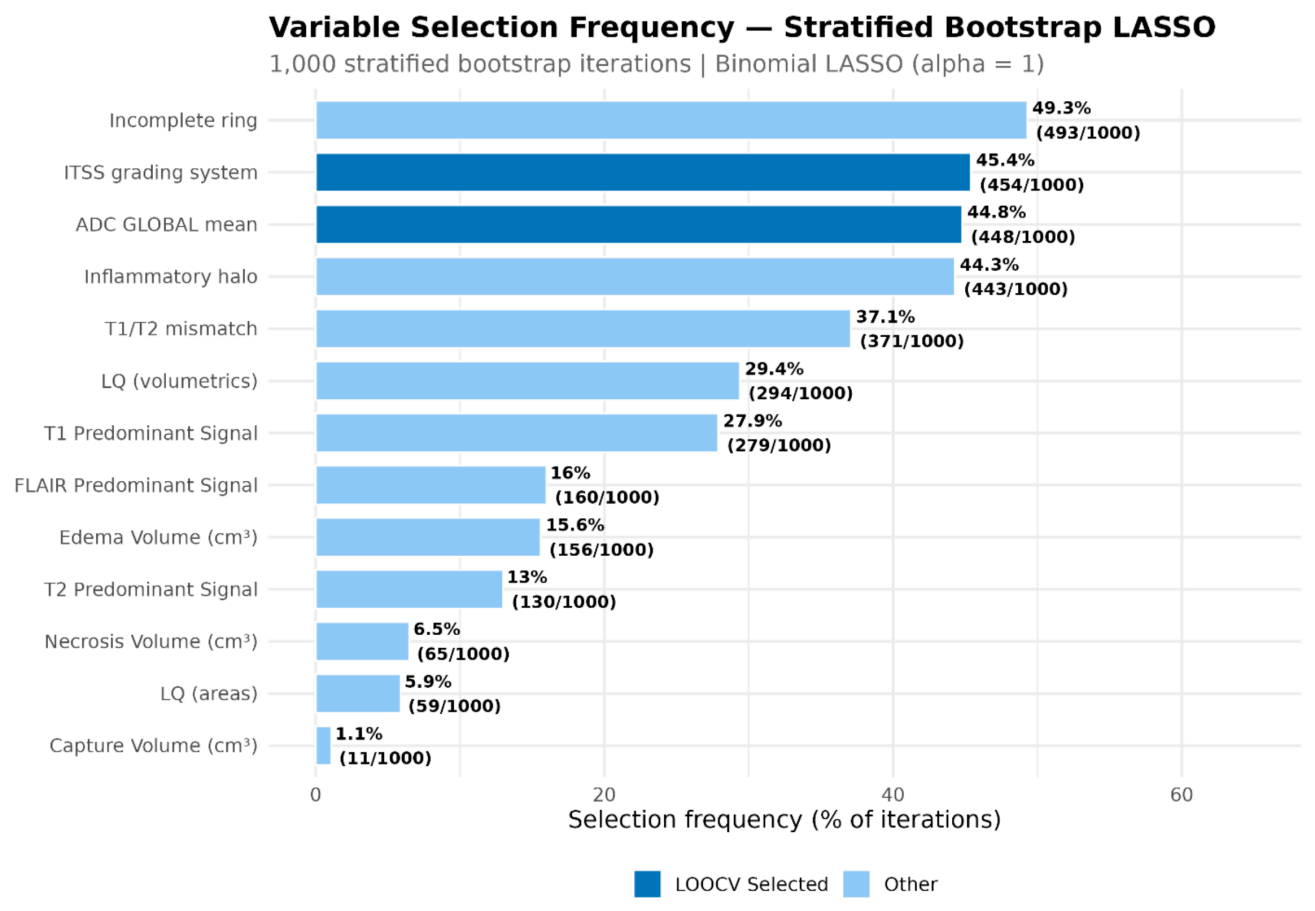
Results of stratified bootstrap analysis (B = 1000). Four variables showed the highest selection frequencies—incomplete ring (49.3%), ITSS grading system (45.4%), mean global ADC (44.8%), and inflammatory halo (44.3%)—identifying them as the most robust predictors of radionecrosis. In contrast, volumetric features were consistently shrunk to zero, indicating limited independent predictive value.

Four features demonstrated the highest selection stability, as shown in Figure 6.

## 4. Discussion

In this study, we investigated the ability of conventional brain MRI to differentiate RN from rM1 in patients who underwent surgical resection followed by adjuvant radiotherapy. Importantly, all cases were histologically confirmed after reoperation, providing a robust reference standard. To our knowledge, this represents one of the few imaging studies specifically focused on post-surgical patients treated with radiotherapy, which is a subgroup in which imaging interpretation is particularly challenging.

With SRS becoming a pivotal therapeutic option for brain metastases and its indications continuously expanding, RN has emerged as a relatively common side effect in clinical practice. MRI plays a key role in the follow-up of these patients, particularly in evaluating treatment response and detecting disease progression. Accurate differentiation between these two entities is desirable [12], as their management strategies and prognosis differ significantly.

However, conventional MRI findings often overlap between the two entities [12,13,14], leading to diagnostic uncertainty and potential mismanagement. This diagnostic overlap was also highlighted by Kim et al. [15], who reported that 89% of patients with histologically confirmed RN were preoperatively diagnosed with recurrent or refractory brain metastases despite previous radiological responses to radiotherapy in most cases. While advanced MRI techniques, such as perfusion imaging and MR spectroscopy, have been proposed to enhance diagnostic accuracy, their routine clinical application remains limited due to longer acquisition times, restricted availability, interobserver variability and susceptibility to artifacts [6,16], particularly in post-surgical patients.

Our results support the continued clinical relevance of standard MRI protocols by identifying a limited set of qualitative and quantitative imaging features that may help guide diagnostic differentiation.

Regarding conventional signal characteristics, T2 SI showed a significant difference between the RN and rM1 lesions with RN displaying a more hyperintense signal due to predominance of necrotic tissue and loss of cellular integrity. In contrast, rM1 lesions typically showed lower T2SI due to greater cellularity and more compact tissue architecture. No statistically significant differences were observed on T1w and FLAIR images, which was consistent with previous reports suggesting that the basic structural appearance on these sequences are often nonspecific [17].

The evaluation of T1/T2 mismatch was not significantly different between the two groups, although rM1 lesions tended to exhibit well-correlated boundaries on T2w and on post-contrast T1w. This result aligns with previous studies that questioned the reproducibility and diagnostic reliability of this sign [17], highlighting its subjective nature and dependence on lesion’s morphology and observer experience.

Leeman et al. [14] reported that the greater edema and edema/lesion ratio on T2w was associated with RN. However, in our analysis, neither edema extension nor the volume of enhancing components and necrosis differed significantly between groups, suggesting that these imaging characteristics may not reliably distinguish the two groups [17].

Regarding enhancement patterns, a peripheral enhancing mass with ill-defined borders and internal soap bubble-like or Swiss cheese-like areas of necrosis has been described as more indicative of RN instead of rM1 [18]; however, the subjective evaluation of these signs limits their reproducibility [19]. In our analysis, peripheral non-linear enhancement was observed exclusively in RN, although other types of contrast enhancement were also present. Conversely, rM1 lesions showed a wider range of enhancement patterns but none exhibited peripheral non-linear enhancement. While enhancement pattern alone lacks sufficient specificity, its integration with other qualitative features may contribute to diagnostic confidence. Beyond conventional enhancement patterns, delayed contrast-enhanced MRI has been investigated as a complementary approach to differentiate RN from tumor recurrence. However, preliminary findings suggest an overlap between TRAMs (Treatment Response Assessment Maps) patterns in the two conditions, highlighting the need for the further optimization and validation of delayed contrast imaging protocols [20].

We also analyzed two recently described qualitative features, the “inflammatory cloud” and “incomplete ring enhancement”, both of which were observed exclusively in RN lesions. These findings, first reported by Ortiz de Mendivil et al. [10], have been attributed to the inflammatory infiltration and demyelination associated with radiation-induced injury. 

In our cohort, the absence of these features in rM1 and their consistent presence in RN suggest that they may represent valuable imaging markers detectable on standard MRI protocols. Importantly, their identification does not require advanced sequences or dedicated post-processing, which enhances their potential applicability in routine clinical practice. Nevertheless, given the limited sample size, these findings should be interpreted with caution and validated in larger cohorts.

Quantitative imaging analysis revealed statistically significant differences in ADC values and ITSS grading between RN and rM1. RN showed higher ADC values, reflecting facilitated diffusion, due to liquefaction and increased water content in interstitial spaces, whereas rM1 exhibited lower ADC values consistent with high cellularity [21].

However, the discriminatory value of ADC may vary across clinical settings. Detsky et al. found no significant difference in ADC between radiation necrosis and tumor recurrence in a histopathologically validated cohort, whereas the IVIM-derived perfusion fraction showed significant discriminatory ability [22]. These findings suggest that perfusion-related parameters may provide complementary information to diffusion metrics, although further validation in larger cohorts is required.

Unlike previous studies that relied on focal or mean ADC measurements [23], our volumetric approach allowed a more comprehensive assessment of diffusion properties across the enhancing lesion, potentially improving robustness and reproducibility. To our knowledge, volumetric ADC evaluation in this specific clinical scenario has not been previously reported.

Similarly, ITSS grading was higher in RN lesions. Radiation-induced vascular damage and hemorrhagic remnants likely account for the increased susceptibility signals observed on SWI in RN. Although ITSS evaluation has been explored in glioma imaging [24], no data are currently available comparing ITSS grading between RN and rM1 in treated brain metastases. Our findings suggest that ITSS grading may serve as an additional quantitative marker, as patients with RN consistently demonstrated higher ITSS grades compared to those with rM1.

We also explored vLQ as an alternative to the classic area-based lesion quotient described in the literature by Desqueada [12] reporting high negative predictive values for selected thresholds. However, other studies [25] showed that LQ was not reliable enough to consistently distinguish between the two entities. In our study, RN lesions demonstrated significantly lower vLQ values compared (0.2) with rM1 (0.65), reflecting the predominance of necrotic components and lack of solid tumor tissue. By incorporating volumetric segmentation, vLQ may better account for the diffuse and irregular morphology typical of RN, potentially reducing subjectivity and improving reproducibility compared with two-dimensional measurements. However, despite statistical significance in univariate analysis, volumetric measures were less frequently retained in multivariate modeling.

The application of penalized regression (LASSO) with LOOCV allowed us to explore multivariable modeling while mitigating the limitations imposed by a small sample size. The overall model performance was moderate (AUC: 0.71, 95% CI: 0.39–1.00), which is consistent with the expected variability in small-sample studies; in small oncological cohorts, machine learning requires safeguards against overfitting [26] and a comprehensive reporting of diagnostic accuracy metrics [27]. Radiomics-based machine learning has also been investigated for differentiating RN from tumor recurrence after SRS, highlighting the growing interest in quantitative MRI approaches for this diagnostic challenge [28]. ITSS grading and ADC values emerged as the most consistently selected predictors across LOOCV iterations, both showing a positive association with RN. Importantly, stability analysis identified four features—incomplete ring enhancement, inflammatory cloud, ADC values, and ITSS grading—as the most consistently selected predictors of RN across bootstrap iterations. In contrast, volumetric parameters were frequently shrunk to zero, suggesting limited independent predictive value when considered alongside qualitative and microstructural features. These findings underscore the importance of integrating visually assessable imaging signs with biologically meaningful quantitative metrics. Given the exploratory nature of this cohort, prospective multicenter validation will be valuable before clinical application [29].

Recent studies have explored multiparametric MRI approaches to differentiate radionecrosis from recurrent brain metastases. In particular, Park et al. [30] investigated structural and physiologic habitat imaging in a larger pathology-validated cohort, highlighting the potential of integrating complementary imaging biomarkers. Our findings complement this approach by identifying conventional MRI features, including the inflammatory cloud and incomplete ring enhancement, alongside ADC values and ITSS grading, that may assist diagnostic assessment in routine clinical practice.

This study has some limitations that should be acknowledged. First, it is a single-center retrospective review conducted by two radiologists, which may affect reproducibility despite consensus reading. A potential limitation of this study is the exclusion of mixed lesions. However, this approach enabled the evaluation of histopathologically unequivocal cases, strengthening the imaging–pathology correlation and minimizing the confounding effect of coexisting viable tumor and radionecrosis. Another limitation is the relatively small cohort size, resulting from the stringent requirement for histopathological confirmation, which is only available in patients undergoing surgical re-operation. Nevertheless, this criterion also represents a major strength, as histopathological assessment remains the gold standard for distinguishing radionecrosis from tumor recurrence. Furthermore, pathology-confirmed datasets in patients with brain metastases are scarce, and few imaging studies have been validated against histological diagnosis, supporting the robustness and clinical relevance of our findings. Additionally, detailed radiotherapy parameters (stereotactic prescription dose in Gy, BED, PTV contours) and concurrent systemic therapies were not systematically available in this retrospective imaging dataset, and one patient contributed two lesions (although patient-level sensitivity analysis confirmed that this did not alter our statistical conclusions).

Larger, multicenter studies would be necessary to validate and refine the imaging features we identified as potential discriminators between RN and rM1, laying the groundwork for future research in this area.

## 5. Conclusions

This study suggests that a standard structural MRI protocol can provide valuable exploratory information to aid in the differentiation between radionecrosis and recurrent brain metastases in post-surgical patients treated with radiotherapy. A limited set of qualitative features—particularly the “inflammatory cloud” and “incomplete ring enhancement” signs—together with quantitative markers such as ADC values and ITSS grading—emerged as the most robust predictors associated with radionecrosis. However, given the small sample size and wide confidence intervals, predictive multivariable models must be regarded as preliminary proof-of-concept analyses. These accessible imaging features may help reduce diagnostic uncertainty in routine clinical practice and should be further validated in larger, multicenter studies with standardized radiotherapy records before clinical implementation.

## Figures and Tables

**Table 1:** Qualitative characteristics of lesions.

	RN		rM1		
Characteristics	No/N Total	%	No/N Total	%	*p*-Value (FDR q)
T1 SI ✓ Hyperintense ✓ Isointense ✓ Hypointense	0/11 2/11 9/11	0% 18% 82%	0/6 3/6 3/6	0% 50% 50%	0.41 (q = 0.447)
T2 SI ✓ Hyperintense ✓ Isointense ✓ Hypointense	8/11 3/11 0/11	73% 27% 0%	1/6 1/6 4/6	17% 17% 66%	0.007 (q = 0.072)
FLAIR SI ✓ Hyperintense ✓ Isointense ✓ Hypointense	0/11 7/11 4/11	0% 64% 36%	1/6 3/6 2/6	17% 50% 33%	0.37 (q = 0.444)
T1/T2 mismatch ✓ Yes ✓ No	9/11 2/11	82% 18%	2/6 4/6	33% 67%	0.11 (q = 0.165)
Inflammatory cloud ✓ Yes ✓ No	7/11 4/11	64% 36%	0/6 6/6	0% 100%	0.034 (q = 0.072)
Incomplete ring enhancement ✓ Yes ✓ No	7/11 4/11	64% 36%	0/6 6/6	0% 100%	0.034 (q = 0.072)

**Table 2:** Different enhancement patterns.

	RN		rM1	
Enhancement Patterns	No/N Total	%	No/N Total	%
Peripheral non-linear enhancement	7/11	64%	0/6	0%
Peripheral	2/11	18%	2/6	33%
Homogeneous	1/11	9%	2/6	34%
Heterogeneous	0/11	0%	2/6	33%
Patchy	1/11	9%	0/6	0%

**Table 3:** Quantitative characteristics of lesions.

*Characteristics*	*RN*	*rM1*	*p*-Value (FDR q)
Enhancing volumes (cm^3^) ✓ Median ✓ IQR	2.84 5.51–1.57	0.98 3.4–0.92	0.25 (q = 0.333)
Necrosis volumes (cm^3^) ✓ Median ✓ IQR	0.27 0.88–0.15	0.09 1.5–0.03	0.59 (q = 0.590)
Edema extension (cm^3^) ✓ Median ✓ IQR	32.12 56.8–19.7	2.78 34.1–2.3	0.09 (q = 0.154)
ADC values ✓ Median ✓ IQR	1.4 1.4–1.1	1 1.2–0.8	0.036 (q = 0.072)
ITSS grading system ✓ Median ✓ IQR	Grade 2 3–2	Grade 1 1–0	0.018 (q = 0.072)
Volumetric LQ ✓ Median ✓ IQR	0.2 0.5–0.1	0.65 0.8–0.5	0.021 (q = 0.072)

## Data Availability

The data supporting the findings of this study are not publicly available due to patient privacy and ethical restrictions. Data may be made available by the corresponding author upon reasonable request, subject to institutional policies and applicable ethical approvals.

## Supplementary Materials

The following supporting information can be downloaded at https://www.mdpi.com/article/10.3390/cancers18213439/s1, Table S1: Diagnostic Performance & Sensitivity Analysis of Predictive Model.

## Abbreviations

The following abbreviations are used in this manuscript:

ADC	Apparent Diffusion Coefficient
AUC	Area Under the ROC Curve
BED	Biologically Effective Dose
FDR	False Discovery Rate
IQR	Interquartile Range
ITSS	Intratumoral Susceptibility Signal
IVIM	Intravoxel Incoherent Motion
LASSO	Least Absolute Shrinkage and Selection Operator
LOOCV	Leave-One-Out-Cross-Validation
LOPO-CV	Leave-One-Patient-Out Cross-Validation
PSP	Pseudoprogression
PTV	Planning Target Volume
rM1	Recurrent Brain Metastasis
RN	Radionecrosis
ROC	Receiver Operating Characteristics
SI	Signal Intensity
SRS	Stereotactic Radiosurgery
SRT	Stereotactic Radiotherapy
TRAMS	Treatment Response Assessment Maps
vLQ	Volumetric Lesion Quotient
WBRT	Whole Brain Radiotherapy
