# Supplementary material for: Structural Magnetic Resonance Imaging (MRI) in Differentiating Radionecrosis from Metastatic Recurrence in Post-Surgical Patients: A Tertiary Center Experience
Source: Cancers (Basel). 2026 Oct 24;18(21):3439. doi: 10.3390/cancers18213439 (PMC13761551; doi:10.3390/cancers18213439)
Supplement: Supplementary file 1 [file cancers-18-03439-s001.zip › cancers-4573300-supplementary.pdf]

Supplementary Material

Structural Magnetic Resonance Imaging (MRI) in Differentiating Radionecrosis from Metastatic Recurrence in Post-Surgical Patients: A Tertiary Center Experience

Journal: Cancers (MDPI)

Table S1: Diagnostic Performance & Sensitivity Analysis of Predictive Model

**Metric**	**Point Estimate**	**95% CI**	**Validation Method**	**Key Finding**
Continuous AUC-ROC	0.71	[0.39, 1.00]	Leave-one-out cross-validation	Promising discriminative performance
Step-Curve AUC-ROC	0.73	[0.36, 1.00]	Monotonic empirical integration	Consistent discriminative ability
Overall Accuracy	76.5% (13/17)	[52.7%, 90.4%]	Optimal Youden threshold (0.609)	Balanced classification (13/17)
Sensitivity (RN)	81.8% (9/11)	[52.3%, 94.9%]	Optimal Youden threshold	9 of 11 radionecrosis identified
Specificity (rM1)	66.7% (4/6)	[30.0%, 90.3%]	Optimal Youden threshold	4 of 6 metastases identified
Fold-Nested Accuracy	76.5% (13/17)	[52.7%, 90.4%]	Threshold nested in each training fold	Robust against threshold selection bias
Patient-Level AUC (LOPO)	0.71	Range: [0.68, 0.73]	Leave-one-patient-out grouping	Robust against clustered lesion data (acc: 75.3%)

Note: 95% confidence intervals (CI) for accuracy, sensitivity, and specificity were calculated using the Wilson score method. Continuous AUC-ROC 95% CI was calculated using DeLong's method. Step-curve AUC 95% CI was computed using stratified bootstrap resampling (2,000 iterations). Fold-nested accuracy was determined by deriving the optimal Youden threshold on n-1 cases and evaluating it on the held-out validation case. Patient-level cross-validation accounts for within-subject correlation from the patient who contributed two lesions.
